# *In Situ* Study of Axial GaSb/GaAs Nanowire Heterostructure Formation

**DOI:** 10.1021/acsnanoscienceau.5c00015

**Published:** 2025-04-15

**Authors:** Mikelis Marnauza, Robin Sjökvist, Azemina Kraina, Daniel Jacobsson, Kimberly A. Dick

**Affiliations:** † Centre for Analysis and Synthesis and NanoLund, 5193 Lund University, 22100 Lund, Sweden; ‡ National Centre for High Resolution Electron Microscopy, Lund University, 22100 Lund, Sweden

**Keywords:** in situ TEM, heterostructures, GaSb, GaAs, III−V semiconductors, nanowires

## Abstract

Combining multiple III–V materials into axial nanowire heterostructures has enabled the fabrication of custom nanowire-based devices useful for a wide range of applications. However, our ability to form axial heterostructures between arbitrary combinations of III–V compounds is impeded by a lack of information on the dynamics of the heterojunction formation process, often resulting in suboptimal heterostructure morphologies, particularly for materials including Sb. In this work, we utilize environmental transmission electron microscopy to examine the formation of GaSb/GaAs heterojunctions in Au-seeded nanowires *in situ*. We demonstrate that the growth parameter window for successful GaSb/GaAs heterostructure formation is very narrow and requires the growth of a ternary GaSb_*x*_As_1–*x*_ segment. Furthermore, we show that as the nanowire changes the composition from GaSb to GaAs, the nanoparticle and nanowire morphologies are highly dynamic. At the end of the transition, we observe that the nanoparticle volume is halved and the nanowire diameter is reduced from ≈40 to ≈30 nm at the liquid–solid interface. Moreover, the nanowire growth rate increases by a factor of 7, when GaAs composition is reached, at our optimized growth conditions. Additionally, we are able to observe that the change in the crystal phase from GaSb zincblende (ZB) to GaAs wurtzite (WZ) happens via a mixed ZB-4H-WZ regime and is dependent not only on the nanowire composition but also on the vapor-phase composition in the growth chamber. These results offer unique insight into the formation dynamics of axial nanowire heterostructures, elucidating the interplay between all phases and growth species.

## Graphical abstract

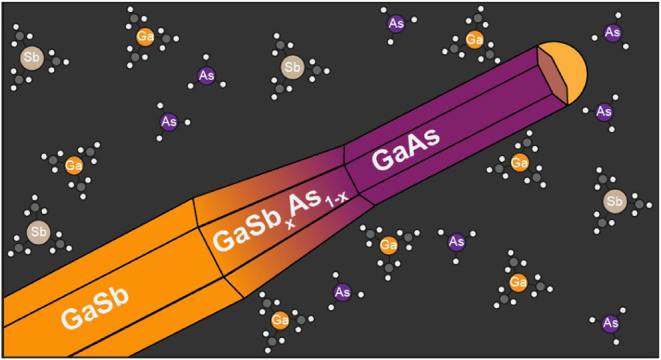

The vapor–liquid–solid (VLS) method is one of the most frequently used techniques for the growth of III–V semiconductor nanowires. Here, solid nanoparticles (most commonly Au) residing on a substrate are exposed to vapor-phase precursors. Thereafter, group III and group V species start to alloy with the metal nanoparticles, resulting in a phase change of the seed nanoparticle from solid to liquid. Eventually, the seed nanoparticles can become supersaturated, which can result in nucleation and growth of nanowires, provided that the precursor supply and suitable substrate temperature are maintained. This allows for the growth of high-compositional-purity binary and ternary III–V compounds that can be used in a variety of applications such as quantum transport, thermoelectrics, and optoelectronics to name a few.^1,2^ However, the real advantage of these nanowires lies in the ability to combine them into core–shell and axial heterostructures, where the combination of semiconductors enables new functionalities.^3^ This is made possible due to the efficient strain relaxation and formation of epitaxial heterostructures between lattice-mismatched materials.^3,4^

Perhaps one of the most challenging axial nanowire heterostructures to grow are the III-Sb/III-As heterostructures (where the heterostructure is formed by changing nanowire composition from an Sb-rich segment to an As-rich segment). In part, this is due to the large lattice mismatch between materials (as is the case in InSb/InAs and GaSb/GaAs heterostructures), but more importantly, this is caused by the complex behavior of elemental Sb.^5^ Antimony is known to behave as a surfactant, altering the surface energies of materials, in addition to having a lower vapor pressure and higher solubility in Au in comparison to As solubility in Au.^2,6,7^ These material-specific properties have a profound effect on our ability to form heterostructures, often making it impossible to reach pure III-As composition once Sb has been introduced to the growth chamber.^8–11^ In the literature, this is summarized by two different phenomena. First, the reservoir effect is caused by the high solubility of Sb in the Au nanoparticle during growth. In axial nanowire heterostructures where III-Sb growth precedes the growth of a different composition segment, such as III-As, this can prevent growth of compositionally pure, Sb-free segments.^8–11^ Additionally, Sb is notorious for contaminating the growth reactors. This effect is termed the memory effect and can be understood as retention of Sb (or Sb-based compounds) on surfaces of the growth system, preventing the growth of Sb-free III–V compounds, therefore, hindering our ability to form III-Sb/III-As heterojunctions.^2,6^ The memory effect is especially pronounced when Sb-rich segments are desired, such as in the case of InSb/InAs and GaSb/GaAs heterostructures. Developing a fundamental understanding of the formation of axial III-Sb/III-As nanowire heterostructures is not only one of the challenges for future nanowire-based device fabrication but also an important step in gaining new insights into nanowire growth involving III-Sb materials.

To address existing gaps in our knowledge of fundamental nanowire growth process, *in situ* studies have been used to great success using specialized environmental transmission electron microscopes (ETEMs). Originally elucidating on the growth of Au-seeded Si nanowires, these studies have, in the recent years, expanded to encapsulate a wide range of III–V nanowires such as GaAs, GaSb, GaP, and InGaAs.^7,12–14^ This has allowed us to expand our understanding of the interplay between vapor, liquid, and solid phases, demonstrating how growth conditions affect the crystal phase and nanowire morphology as well as showcasing departure from the layer-by-layer growth mode in favor of the more complex multilayer growth.^15–20^

In this study, we investigate the formation of axial GaSb/GaAs nanowire heterostructures. This is achieved by conducting the nanowire growth process *in situ* using an ETEM. We investigate how precursor balance and different switching procedures affect the heterostructure morphology, highlighting that the growth parameter window for successful GaSb/GaAs heterostructure formation is very narrow and requires the growth of a ternary GaSb_*x*_As_1–*x*_ segment, which in this study was ≈ 50 nm long. By acquiring high frame rate videos of the heterostructure formation process, we demonstrate the dynamics of nanowire and nanoparticle morphology as well as the dynamics of the growth rate and crystal phase, and how they relate to precursor supply. When switching from GaSb to GaAs, we observe that the nanoparticle volume is halved (which resulted in nanowire diameter change from ≈ 40 to ≈ 30 nm at the liquid–solid (LS) interface) and the growth rate is increased by a factor of 7 at our optimized growth conditions. Furthermore, we show that a change in the crystal structure, from zincblende (ZB) to wurtzite (WZ) via a mixed ZB-4H-WZ structure, happens after steady-state growth of GaAs is achieved and is highly dependent on the gas-phase composition in the growth chamber.

## Results and Discussion

### Overview of the GaSb/GaAs Heterojunction Formation

Before examining the details of GaSb/GaAs heterostructure formation, we start by briefly discussing the *in situ* growth of GaSb nanowires. To facilitate the nanowire growth process, Au nanoparticles with nominal diameters of 30 nm were used. The growth is performed by supplying trimethylgallium (TMGa) and trimethylantimony (TMSb), with H_2_ as the carrier gas for both precursors, to the ETEM. The growth temperature for GaSb nanowires and throughout heterostructure formation was set to 420 °C. The specific partial pressures used for the precursors along with additional details of the growth are given in the Methods section. In this study, the nucleation of GaSb nanowires is performed directly on the amorphous SiN_*x*_ from Au nanoparticles. Due to the amorphous nature of the substrate, the nanowires shortly after the nucleation stage are highly dynamic until a stable {111} growth front is established.^21^ This can result in the base of nanowires often having larger dimensions and stacking defects before steady-state growth is established.

When steady-state growth of GaSb nanowires is attained, we can turn our attention to heterostructure formation. The intended axial nanowire heterostructure is shown in Figure 1a, where a ternary segment of GaSb_*x*_As_1–*x*_ was utilized to enable switching, with a schematic of the growth precursor supply partial pressures shown in Figure 1b. Here, we can see that to initiate the heterojunction formation and growth of the ternary GaSb_*x*_As_1–*x*_ segment, TMGa and TMSb are supplied to the microscope concurrently with arsine (AsH_3_) for 60 s. Thereafter, the TMSb supply is stopped, and AsH_3_ partial pressure is increased. Meanwhile, the TMGa supply throughout the process is kept constant. For detailed partial pressures of the precursors and H_2_ during the heterostructure formation, please see Supporting Information SI-1.

**Figure 1:**
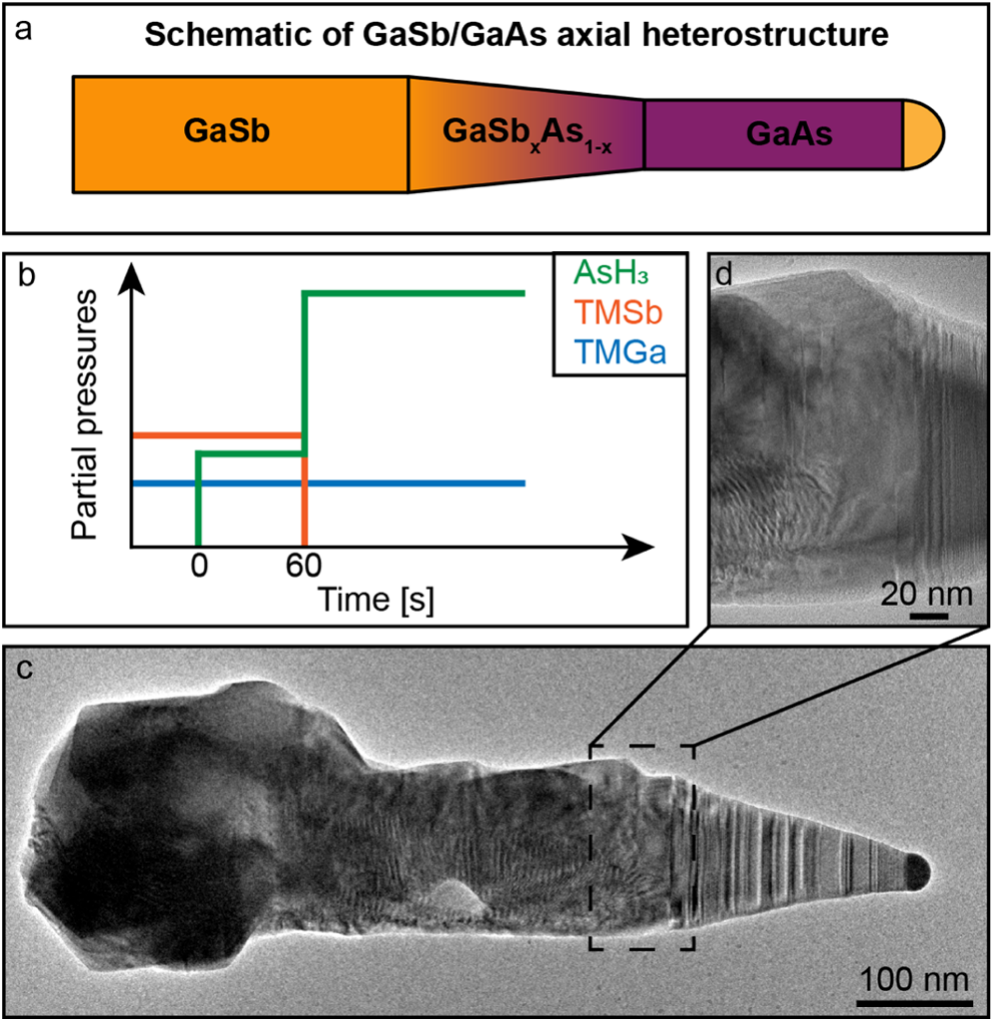
GaSb/GaAs heterostructure overview. (a) Schematic overview of the intended GaSb/GaAs axial nanowire heterostructure. (b) Schematic overview of the precursor balance during the heterojunction formation. (c) HRTEM image of a GaSb/GaAs nanowire after the switch. (d) Close-up image of the transition region between GaSb and GaAs from panel (c). The strain fields in the image arise due to As-rich overgrowth on the initial GaSb stem.

The combined intermittent supply of AsH_3_ and TMSb facilitates the growth of a short GaAs_1–*x*_Sb_*x*_ segment, which, as will be discussed in section Kinking/Change of Growth Direction, is crucial to avoid the kinking process and allow continued axial growth of the heterostructure. The growth conditions of the GaAs segment (following the growth of the ternary segment) were chosen to support the growth of WZ GaAs.^15,17,18^ This, combined with the fact that the growth of GaSb nanowires always occurs in a ZB crystal phase, allowed us to indirectly track the compositional change occurring in the nanowire as well as provide information on crystal-phase selection during heterostructure formation.^22^ This will be discussed in more detail in the section Crystal-Phase Stability.

A post-growth high-resolution transmission electron microscope (HRTEM) image of the complete GaSb/GaAs nanowire heterostructure is demonstrated in Figure 1c. Throughout the middle part of the heterostructure, shown in Figure 1c, the presence of strain fields can be observed, indicating a strained core–shell structure. Additionally, moiré fringes can be seen, which are expected to arise due to an As-rich solid overgrowing the initial GaSb nanowire core. By observing Video S1 and comparing images before and after switching that can be viewed in Supporting Information SI-2, it is evident that the original GaSb nanowire has undergone significant overgrowth of As-rich solid, which is also confirmed by post-growth X-ray energy dispersive spectroscopy (XEDS) mapping (see Supporting Information SI-3). In Figure 1d, a close-up HRTEM image of the GaSb/GaAs heterojunction is shown. Here, it is possible to observe the gradual suppression of strain fields and change in the crystal structure from pure ZB to increasingly longer segments of pure WZ via the mixed ZB-4H-WZ crystal phase.

### Dynamics of the GaSb/GaAs Heterojunction Formation

To investigate the dynamics of heterojunction formation, we analyzed *in situ* videos of the heterojunction formation. This enabled a much more detailed analysis of changes in the growth rate, morphology, and general layer-by-layer growth dynamics than conventional post-growth analysis of nanowire heterostructures. Furthermore, tracking parameters such as the nanoparticle volume and nanowire growth rate allows us to indirectly obtain information on the thermodynamics and kinetics that occur during heterojunction formation. In this section, we will analyze the heterojunction formation depicted in Video S1 at 16× original speed. In this video, the timestamp is set relative to the onset of AsH_3_ supply, meaning that 0 s marks the time when AsH_3_ is supplied to the microscope in accordance with the schematic in Figure 1b.

The data presented in Figure 2 depict changes in the nanoparticle and nanowire as it transitions from the steady-state growth of GaSb to the steady-state growth of GaAs. In Figure 2a, a GaSb nanowire during the steady-state growth is displayed before heterojunction formation is started. From the inset containing a fast Fourier transform (FFT) pattern of the nanowire, we can determine that the GaSb nanowire is ZB-aligned to the [11̅0] zone axis. The timestamp in the bottom left corner indicates that the image is captured 18 s before AsH_3_ is supplied to the microscope. The red dashed outline surrounding the nanoparticle is used as a guide to the eye in order to illustrate the original nanoparticle dimensions before switching to GaAs growth. Figure 2b depicts changes in the nanoparticle volume, nanowire growth rate, and (111)/(0002) lattice spacing as a function of time during the switching process. The (111)/(0002) lattice planes are perpendicular to the growth direction in ZB and WZ, respectively. By measuring the (111)/(0002) lattice spacing, we are able to observe the compositional change of the nanowire solid via a ternary GaSb_*x*_As_1–*x*_ segment, in accordance with Vegard’s law.^23^ The nanoparticle volume was calculated assuming the nanoparticle to be hemispherical using the methodology described elsewhere.^20,24^ In all of the graphs, the time of 0 s corresponds to initiation of AsH_3_ supply and is marked by dashed line (1), while dashed line (2) indicates the time when the AsH_3_ partial pressure was increased and TMSb supply to the microscope was stopped (as schematically shown previously in Figure 1b).

**Figure 2:**
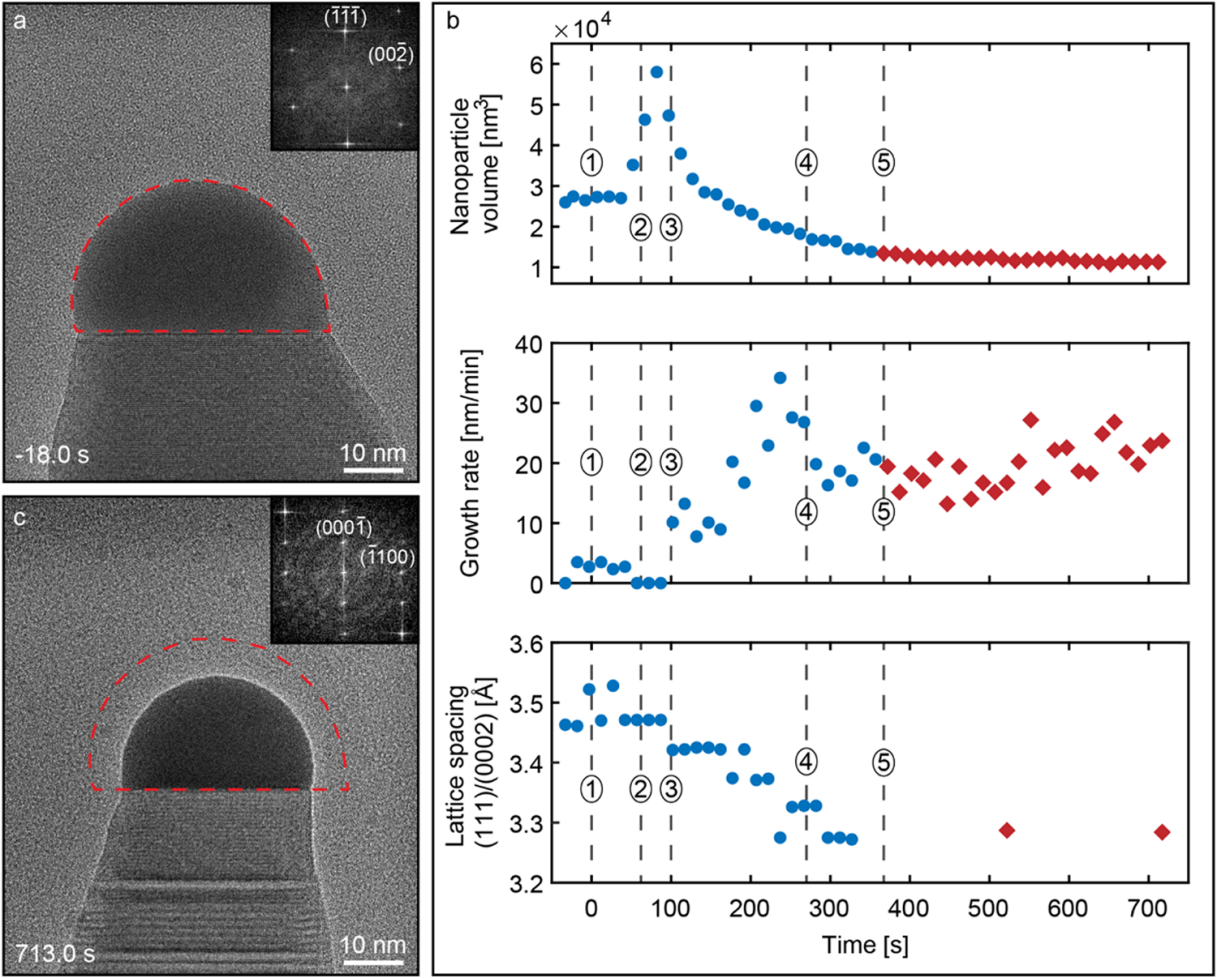
*In situ* data of GaSb/GaAs heterojunction formation. (a) HRTEM image of a Au-seeded GaSb nanowire 18 s before commencing the supply of AsH_3_. Inset shows an FFT of the crystal region near the LS interface, indicating that it is the ZB crystal phase oriented to the [11̅0] zone axis. (b) Change in the nanoparticle volume, growth rate, and (111)/(0002) lattice spacing as a function of time during the heterojunction formation. In the plots, the dashed numbered lines indicate the (1) time when AsH_3_ is supplied to the microscope, (2) AsH_3_ supply is increased/TMSb supply is stopped, (3) nanowire growth rate rapidly increases/lattice spacing decreases, (4) nanowire starts to grow in a twinned ZB crystal structure, and (5) nanowire starts to form WZ segments. Data marked with blue circles and red diamonds indicate that the nanowire grows in a ZB and a 4H-WZ regime, respectively. (c) HRTEM image of a Au-seeded GaAs nanowire during growth 713.0 s after commencing supply of AsH_3_. Inset shows an FFT of the crystal region near the LS interface, indicating that it is WZ oriented to the [112̅0] zone axis. Red dashed outlines in panels (a) and (c) are used as a guide to the eye, depicting change in nanoparticle dimensions when nanowire growth is changed from GaSb to GaAs.

No significant changes can be observed to the growth and morphology until TMSb supply is stopped and AsH_3_ partial pressure is simultaneously increased, as marked by the dashed line (2) in Figure 2b. Hereafter, we observed rapid doubling of the nanoparticle volume (75–100 s). This is seemingly caused by axial growth stopping (which causes material accumulation in the nanoparticle). As can be observed from Figure 2b, this in turn correlates well with the increase in AsH_3_ supply/stopping of TMSb supply to the microscope. The swelling of the nanoparticle is followed by a rapid increase in the nanowire growth rate that causes the beginning of a gradual decrease in the nanoparticle volume around the 100 s mark indicated by the dashed line (3) in Figure 2b. This dramatic increase in the growth rate and decrease in nanoparticle volume is also accompanied by a reduction of the nanowire lattice spacing. Since the lattice spacing of ZB GaSb is larger than that of ZB and WZ GaAs, this indicates that the nanowire composition is shifting toward more As-rich composition.^2,25^

The dramatic change in nanoparticle volume can be explained by fundamental differences in nanoparticle composition during the growth of Au-seeded GaAs and GaSb nanowires. Previous *in situ* studies of Au-seeded GaAs nanowires have shown that the steady-state growth of GaAs generally requires Ga concentrations of approximately 25–55 at.%.^17^ This is in stark contrast to steady-state growth of Au-seeded GaSb where our previous studies show that the Ga concentration in the nanoparticle is generally in the 66–94 at.% range.^7,20^ Therefore, when the nanowire starts to transition to a more As-rich composition, the nanoparticle composition in terms of Ga concentration far exceeds that of the GaAs steady-state growth requirements, resulting in a massive increase in growth rate peaking at ≈34 nm/min.^26^

At around 270 s into the switching process, the nanowire growth rate was observed to stabilize at ≈20 nm/min, which is a nearly 7-fold increase from the GaSb growth rate, indicated by dashed line (4) in Figure 2b graphs. Moreover, at this time, we also observed that the nanowire starts to form ZB twins. The twinning process eventually evolved into nanowire growth with a mixed ZB-4H-WZ crystal phase, subsequently resulting in the growth of WZ segments at approximately 380 s, as indicated by the dashed line (5). Due to frequent changes in the crystal structure, after 350 s, the measurement of lattice spacing was complicated; however, additional measurements at ≈520 and ≈710 s matched the expected (0002) spacing for WZ GaAs. At this point, the nanoparticle volume has stabilized at a volume that is approximately half of the original nanoparticle volume consistent with the lower Ga concentration of Au-seeded GaAs nanowire growth. In Figure 2c, an HRTEM image extracted from the *in situ* video displays the nanowire when steady-state growth of GaAs is established. Here, the red dashed outline is used to illustrate the original dimensions of the nanoparticle before heterojunction formation. Furthermore, when comparing the images in Figure 2a,c, it is evident that the change in nanoparticle volume has resulted in a change in nanowire diameter from about 42 nm (during steady-state GaSb growth) to 32 nm (when steady-state GaAs growth is attained). Additionally, from the inset FFT pattern of the top segment of the nanowire, we can determine that the GaAs nanowire is WZ aligned to the [112̅0] zone axis.

The time scale of the compositional change from steady-state growth of GaSb to steady-state growth of GaAs is relatively long (approximately 350 s). However, as the growth rate of our nanowire is low, it equates to a short ternary segment.^27,28^ This is demonstrated in Figure 3a, where an image of the nanowire during the heterojunction formation process is shown, while a complementary geometric-phase analysis (GPA) color plot is depicted in Figure 3b. Here, the color depicts interplanar lattice spacing for (111) lattice planes with the smaller lattice spacing indicating an As-rich ternary GaSb_*x*_As_1–*x*_ in accordance with Vegard’s law. The boundaries of the GPA color plot were chosen based on theoretically calculated lattice spacings of pure ZB GaSb and ZB GaAs using lattice parameters reported elsewhere.^25,29^ By comparing the real space image in Figure 3a with the GPA-treated image in Figure 3b, it becomes evident that the length of the ternary GaSb_*x*_As_1–*x*_ segment is approximately 50 nm. A 4× sped up *in situ* real space video coupled with GPA encompassing the full compositional switch can be viewed in Video S2.

**Figure 3:**
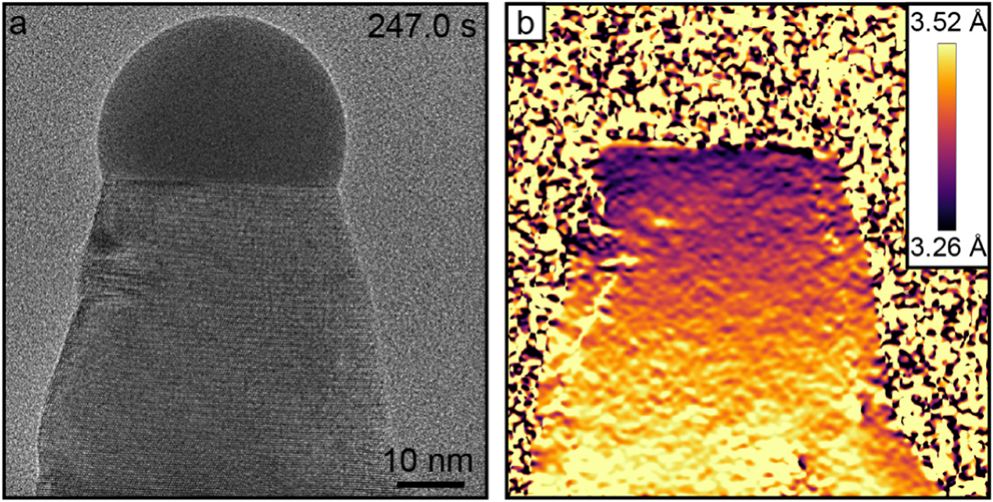
Ternary segment length between GaSb and GaAs. (a) HRTEM image of a Au-seeded GaAs nanowire 247.0 s after commencing the supply of AsH_3_. (b) Same image after GPA treatment, where the color represents lattice spacing for (111) lattice planes with the smaller lattice spacing indicating an As-rich composition.

Based on our observations, the time when TMSb supply is stopped (around the 100 s mark indicated by dashed line (2) in Figure 2b) is the deciding moment on the resulting heterostructure morphology. Up until this point, the nanowire composition is that of pure GaSb, as indicated by the measured lattice spacing; however, as TMSb supply is stopped, nucleation of new layers will have to involve the incorporation of As. The interruption in the axial growth rate following the stop in TMSb supply suggests that it is energetically unfavorable to nucleate As-rich layers on GaSb. Since crystallization is stopped, but material supply from the vapor phase continues, this results in a rapid increase in nanoparticle volume. We speculate that the high energy cost of nucleation of a new material on the grown GaSb is counterbalanced by the extreme driving force for nucleation of As-rich layers that arises due to high Ga concentration in the nanoparticle. From this point, the nanowire can either change its growth direction/kink or continue growing axially resulting in a GaSb/GaAs heterostructure, which we observed to be highly dependent on the vapor-phase composition of the growth chamber. The resulting morphology is discussed in more detail in section Kinking/Change of Growth Direction.

### Crystal-Phase Stability

From Figures 2 and 3, it is apparent that the change in the nanowire crystal structure seemingly “lags” behind morphological and compositional changes in the nanowire. Furthermore, from Figure 2b, it can be observed that twinning in the nanowire begins at the very end of the nanowire compositional change (as indicated by the change in lattice spacing). The twinning regime is subsequently followed by growth in a mixed regime. Here, in addition to ZB twins, we observed a high density of stacking faults intermixed with short segments of the 4H crystal phase. This was followed by growth of WZ segments of increasing frequency and length.

In order to investigate the delayed change in the crystal structure, in addition to nanowire lattice spacing, we studied the gas composition exiting the microscope. This was achieved by analyzing the data extracted from a residual gas analyzer (RGA) attached to the vent line of our ETEM. As the RGA is positioned in the exhaust of the microscope, it does not give an exact measure of the partial pressures at the sample; however, it does provide an indication of the relative pressures of precursors present in the gas phase.^21,30^ In Figure 4a, we show the RGA data plotted as a function of time during the heterostructure formation process until the growth of WZ was observed. Here, similar to Figure 2, dashed line (4) in the plot marks the time when twinning in the nanowire is first observed, while dashed line (5) indicates the time when the growth of prolonged WZ segments was seen. An HRTEM image depicting the nanowire shortly after twinning begins is shown in Figure 4b with the complementary FFT pattern of the region indicated by the blue box shown in Figure 4c. In the FFT pattern, the white and orange circles indicate the diffraction spots of the twinned ZB crystals. Moreover, in Figure 4d, the HRTEM image of the same nanowire shortly after WZ formation was observed is shown, with the green box indicating a region from which a complementary FFT pattern is displayed in Figure 4e. In the FFT pattern, the orange and white circles mark select reflections of 4H and WZ crystal phases to showcase the double periodicity of 4H over the WZ crystal phase.

**Figure 4:**
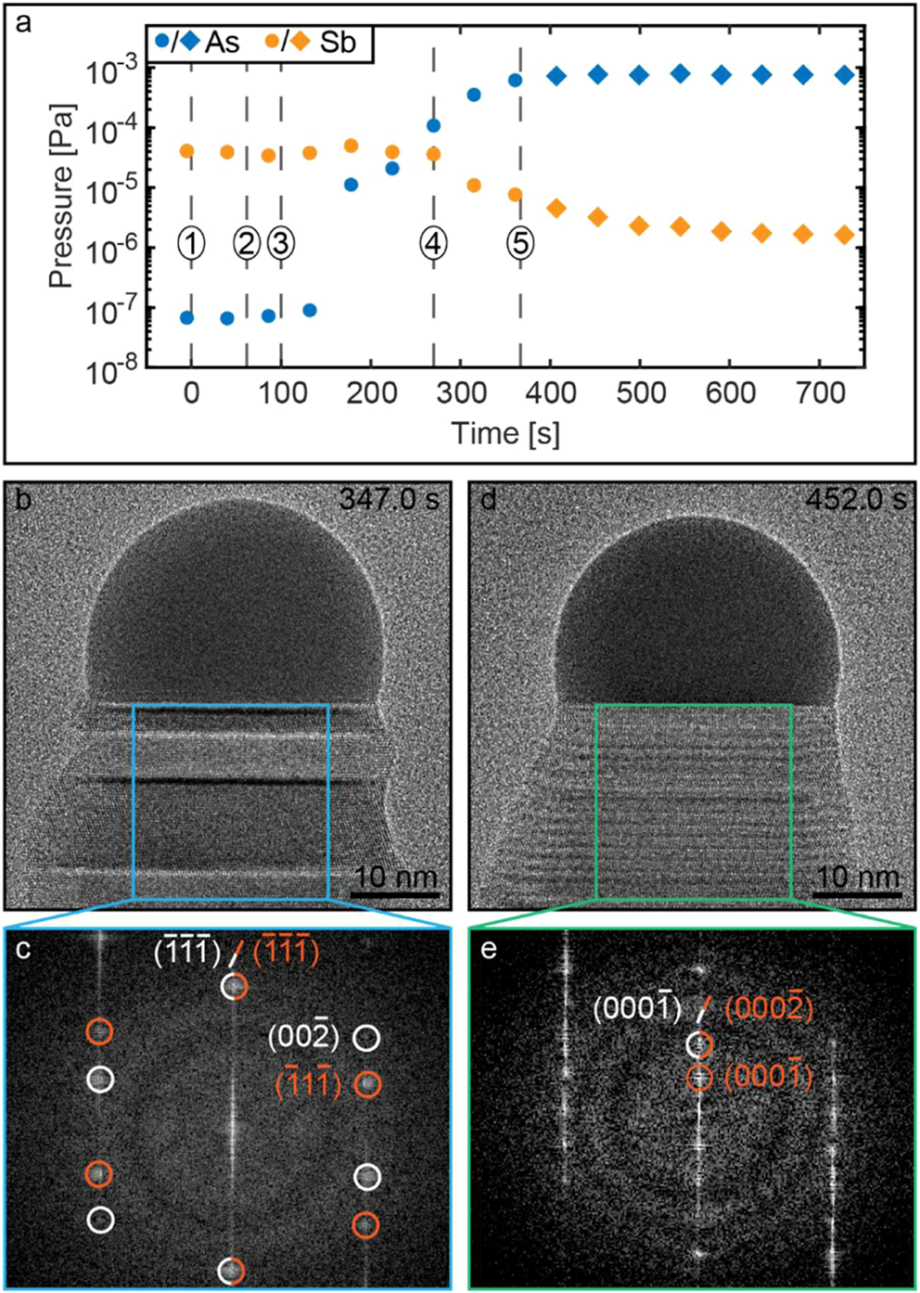
Evolution of the crystal- and vapor-phase composition. (a) RGA pressure data on As and Sb in the vapor phase during heterostructure formation. In the plot, the dashed numbered lines indicate the (1) time when AsH_3_ is supplied to the microscope, (2) AsH_3_ supply is increased/TMSb supply is stopped, (3) nanowire growth rate rapidly increases/lattice spacing decreases, (4) nanowire starts to grow in the twinned ZB crystal structure, and (5) nanowire starts to form WZ segments. Data marked with circles and diamonds indicate that the nanowire grows in a ZB and a 4H-WZ regime, respectively. (b) HRTEM image of an Au-seeded GaAs nanowire 347.0 s after commencing supply of AsH_3_. (c) FFT of the crystal region marked by the blue box in panel (b) revealing a twinned ZB crystal phase. Select reflections for the two rotational twins are highlighted using white and orange circles in the FFT. (d) HRTEM image of a Au-seeded GaAs nanowire 452.0 s after commencing supply of AsH_3_. (e) FFT of the crystal region marked by the green box in panel (d), revealing a predominantly 4H crystal phase. Select reflections for WZ and 4H crystal phases are shown in white and orange, respectively, to show that 4H has double periodicity of WZ.

From the RGA data in Figure 4a, it is clear that the observed rapid increase in growth rate/decrease in lattice spacing marked by the dashed line (3) in Figure 2b corresponds to the point where the As pressure, as measured by RGA, starts to dramatically increase. Furthermore, we observe that twinning begins when the As pressure surpasses that of Sb, as indicated by the dashed line (4) in Figures 2b and 4a. Similarly, the formation of the WZ phase is only observed when the Sb pressure in RGA reaches the minimum measured value, as indicated by dashed line (5) in Figures 2b and 4a. Therefore, it is evident that the change in the crystal structure is affected by the partial pressure of Sb, As, and/or the As/Sb ratio in the vapor phase. These observations are in agreement with previous studies of nanowire heterostructures involving III-As and III-Sb where even small amounts of Sb residues in the growth system affected the crystal phase of nanowires.^8,10,22^ Commonly, the explanation for Sb affecting the nanowire crystal structure is attributed to the surfactant effect that is expected to alter the surface and interfacial energies determining the crystal-phase selection.^2^

Although the onset of the crystal structure change from ZB to a mixed regime coincides well with the RGA data, it is impossible to exclude that trace amounts of Sb (in the range of 1–2 at.%) could still be present in the nanowire or the liquid nanoparticle during the switching process. Such trace amounts could significantly affect the energy balance between the nanoparticle and solid nanowire and would have a negligible impact on nanowire lattice spacing and be below the detection limit of the XEDS system at the used growth temperature.^8,18^

Our observations of ZB being the preferred crystal phase until full compositional switch to GaAs is attained are in line with previous reports involving III-Sb materials.^8,10,11,22,31^ The III-Sb nanowires such as InSb and GaSb in general favor the ZB crystal phase. Ternary segments often exhibit a mixed structure (although this is highly dependent on the Sb concentration in the ternary segment), whereas the WZ crystal phase (of the GaAs) is attainable only at negligible or nonexistent Sb background. Therefore, the crystal phase in III-Sb/III-As heterostructures can be used as an extremely sensitive way to determine the compositional purity.

### Kinking/Change of Growth Direction

Atomically sharp compositional interfaces are often desired in nanowire heterostructures. Therefore, we examined heterojunction formation procedures that focus on minimizing the length of the ternary GaSb_*x*_As_1–*x*_ segment. However, we consistently observed that procedures not involving a brief concurrent supply of TMSb and AsH_3_ resulted in the nanowire changing its growth direction and kinking by Au nanoparticle migration from the liquid–solid (LS) interface. These heterojunction formation procedures can broadly be separated into two main categories: switching with and without residual TMSb in the group V line of our growth system. In the first case, AsH_3_ supply to the ETEM is initiated with a significant concentration of TMSb still in the gas line. On the other hand, in the second case without residual TMSb in the gas line, before AsH_3_ supply is initiated, the remainder of TMSb from the gas line volume is vented into a vent line bypassing the ETEM. For a more detailed description of the ETEM, the readers are referred to the article by Tornberg et al.^32^ It is important to note that in either case during switching, residual TMSb was present in the growth chamber. However, due to additional TMSb in the group V line, the background pressure of TMSb was effectively present for a longer time relative to when AsH_3_ supply was initiated in the first case.

First, we address the switching procedure where the group V line leading up to the ETEM was evacuated of TMSb before initiating the supply of AsH_3_ (details on the group V line venting procedure can be found in the Methods section). An example nanowire before and after this procedure is depicted in Figure 5a. From the images in Figure 5a, it can be seen that the Au nanoparticle leaves the original LS interface and proceeds to etch the existing GaSb nanowire. This destabilization of the nanoparticle was in some cases observed to occur due to dissolution of the as-grown nanowire at the triple-phase line. The etching of the GaSb segment indicates that the chemical potential in the liquid nanoparticle is lower than that of the solid, which forces more and more solidified material to be dissolved. Concurrently, due to evaporation and/or out-diffusion of material from the nanoparticle, this creates a cycle where the nanoparticle continuously etches the GaSb nanowire.

**Figure 5:**
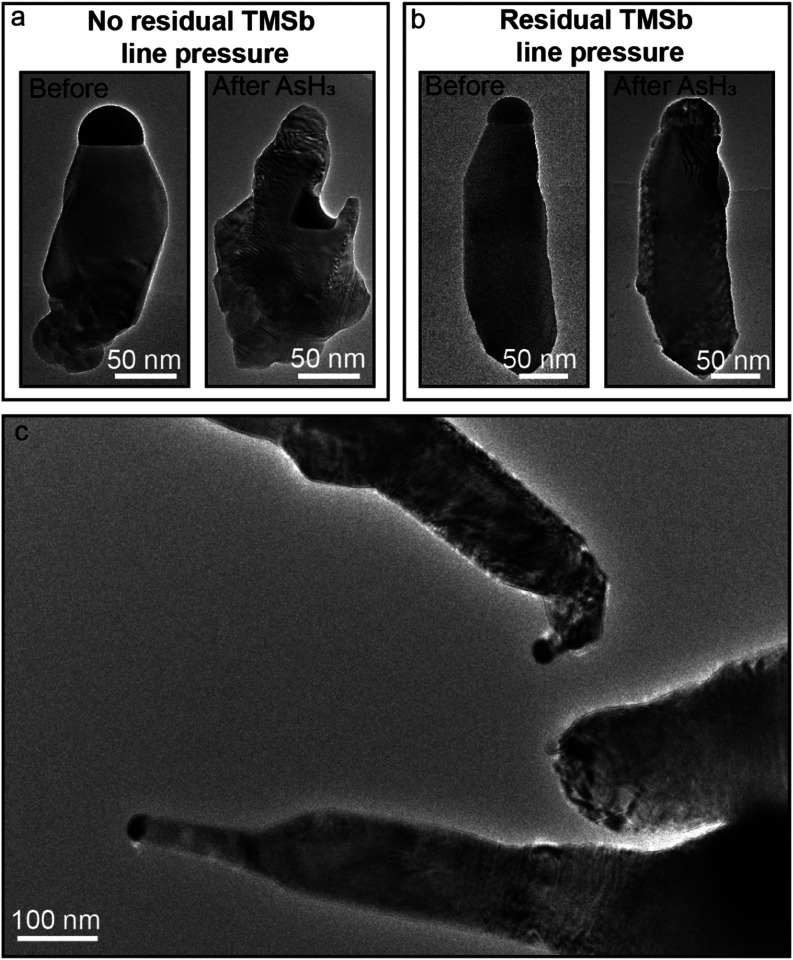
Different approaches to axial GaSb/GaAs nanowire heterostructure formation. (a) Before and after images of a nanowire where the remainder of TMSb was removed from the group V line before a supply of AsH_3_ was initiated. (b) Before and after images of a nanowire where AsH_3_ was supplied to the system without removing TMSb from the group V line. (c) Overview image of nanowires after switching from GaSb to GaAs, showcasing different morphologies of nanowire heterostructures.

A previous *in situ* study of etching in Au-seeded GaAs nanowires described the process to be reliant on the Ga concentration in the nanoparticle.^33^ However, in our experiments, etching of the GaSb nanowire was observed, while TMGa and AsH_3_ were actively supplied to the growth chamber, suggesting that it is the depletion of Sb in the nanoparticle that drives the etching process. In our previous works, we have demonstrated that during steady-state growth of Au-seeded GaSb nanowires, there is a considerable amount of Sb atoms dissolved in the liquid Au–Ga alloy (about 3–4 at.%).^7,20^ Conversely, in steady-state growth of Au-seeded GaAs nanowires, the nanoparticle is expected to contain less than 1 at.% As dissolved into the liquid Au–Ga alloy.^17^ This indicates that the equilibrium concentration of Sb atoms in the liquid Au–Ga alloy is higher than for As atoms, which could lead to etching under the Sb-deficient vapor phase despite active supply of TMGa.

Next, we examine the results of the switching procedure, where the AsH_3_ supply to the ETEM is initiated without evacuating the residual TMSb from the group V line. A representative result of the outcome of such a procedure is depicted in Figure 5b. In contrast to the case where TMSb was evacuated from the group V line (shown in Figure 5a), the Au nanoparticle after toppling from the LS interface does not etch into the nanowire. An overview image of a different experiment utilizing the approach in which residual TMSb in the group V line is used for switching is demonstrated in Figure 5c. Here, it can be observed that while for some nanowires the Au nanoparticle migrates away from the LS interface, other nanowires in near proximity successfully form axial GaSb/GaAs heterostructures. This suggests that a prolonged supply of TMSb to the growth chamber while AsH_3_ supply is commenced has a significant beneficial effect on the nanoparticle stability and allows successful formation of GaSb/GaAs heterostructures as demonstrated in the previous sections of this study.

Regardless of the utilized approach, whenever a change in growth direction/kinking occurred, it was caused by formation of a new nucleus at the LS interface. The nucleus subsequently grew as a 3D island or a multilayer (rather than layer-by-layer), effectively toppling the nanoparticle. The main difference between island and multilayer growth is in the growth dynamics. The 3D crystalline island can be observed to grow along several crystallographic directions simultaneously, while the multilayer stack (apart from nucleating new layers) grows parallel to the LS interface.^16^ An example of kinking initiated by the formation of a 3D crystalline island is showcased in Figure 6a–c. In Figure 6a, the GaSb nanowire is shown before heterojunction formation. Shortly after supplying AsH_3_ to the growth chamber, a crystalline island forms at the LS interface, illustrated by the white arrow in Figure 6b. The island grows in size, eventually displacing the liquid nanoparticle off the LS interface, leading to kinking shown in Figure 6c.

**Figure 6:**
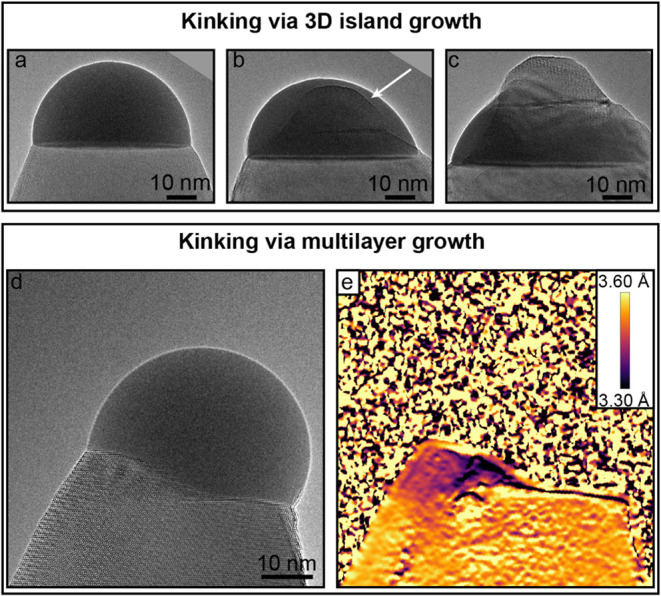
Kinking during heterojunction formation. (a) HRTEM image of the GaSb nanowire before GaSb/GaAs heterojunction formation. (b) During heterojunction formation, a 3D crystalline island forms illustrated by the white arrow. (c) Island grows in size, toppling the liquid seed nanoparticle. (d) HRTEM image of a GaSb nanowire during the GaSb/GaAs switching process with a multilayer stack. (e) GPA-treated image from panel (d). The color represents (111) plane interplanar distance in the growth direction in Å (see the color bar). Smaller spacing indicates more As-rich composition.

On the other hand, in some cases, we were able to observe that this nucleus forms via the multilayer growth mode, which has recently been shown to also cause kinking in the InGaAs nanowire growth.^16^ Kinking due to forming a multilayer stack can be viewed in Video S3, which is 4x sped up and combined with a GPA-treated version of the video. A HRTEM image extracted from Video S3 displaying the multilayer stack is shown in Figure 6d. The GPA analysis of the nanowire growth revealed that the forming multilayer stack exhibits a smaller lattice spacing than the previously grown GaSb, suggesting As incorporation, as shown in Figure 6e. Therefore, nucleation of new As-rich partial layers is energetically more preferential than completion of the partial layers leading to island growth. The kinking of nanowires during heterojunction formation is ultimately a result of the destabilization of the nanoparticle from the growth front. The specific mechanism of kinking (via multilayers, 3D island formation, dissolution/etching of the as-grown nanowire) is strongly dependent on the particular ambient conditions. Contrary to GaSb/GaAs axial heterostructures (III-As segment grown on top of III-Sb), successful growth of GaAs/GaSb heterostructures (III-Sb segment grown on top of III-As), without change of growth direction/kinking, have been demonstrated in previous studies numerous times.^2,7,20,22,27,34^ Therefore, our findings are in line with observations in other axial heterostructure systems where switching is “easier” in one direction than the other due to the interface energy balance between the two materials and the nanoparticle.^4^

## Conclusions

In conclusion, we have investigated the formation of GaSb/GaAs axial heterostructure heterojunctions displaying the dynamics of nanowire and nanoparticle morphology, composition, and crystal phase during the switching process. Our study suggests that the growth parameter window for successful GaSb/GaAs heterostructure formation is very narrow and requires the growth of a ternary GaSb_*x*_As_1–*x*_ segment, which in our case was approximately 50 nm in length. As the nanowire transitions from GaSb to GaAs, we observed that the nanoparticle volume is halved, in turn reducing the nanowire diameter from around 40 to approximately 30 nm. Moreover, changes in the nanowire and nanoparticle morphology were accompanied by a 7-fold increase in the growth rate when the nanowire composition transitioned from GaSb to GaAs at our optimized growth conditions. Furthermore, we showed that the change in the crystal structure from ZB GaSb to WZ GaAs happens via the mixed ZB-4H-WZ structure and is not only dependent on the nanowire but also on the vapor-phase composition in the growth chamber. These findings not only improve our fundamental understanding of heterostructure formation but also provide insight into the variety of factors that need to be considered in order to allow successful growth of large lattice-mismatched and/or antimonide-containing axial nanowire heterostructures.

## Methods

### Imaging and Acquisition of *In Situ* Movies

The nanowire heterostructures were grown inside a Hitachi HF-3300S ETEM equipped with a cold field-emission gun operating at 300 kV. Furthermore, the microscope is equipped with a CEOS BCOR image aberration corrector. The image and *in situ* movies were acquired by using a GATAN OneView IS camera.

### XEDS Acquisition and Analysis

To obtain XEDS spectra and maps of the heterostructures, the microscope is equipped with an Oxford Instruments SDD X-Max^N^ 80T system. The TEM-XEDS spectra were recorded by converging the probe to a 10–20 nm diameter spot and positioning it on the liquid nanoparticle or the as-grown solid nanowire segment. The spectra were recorded with a sampling time of 120 s in a 0–20 keV energy range. For the XEDS mapping of heterostructures, the microscope was operated in the scanning transmission electron microscope (STEM) mode. Quantification of the spectra was carried out using Aztec software (version 3.3) utilizing the Cliff–Lorimer method with the built-in theoretical k-factors.

### Sample Substrate

Microelectromechanical-system (MEMS)-based heating chips supplied by Norcada Inc. were used for *in situ* ETEM growth. The central area of the chip consists of 19 openings surrounded by a thin, electron-transparent SiN_*x*_ membrane onto which the Au nanoparticles seeding the growth are deposited.^35^ The SiN_*x*_ membrane is overlaid onto a tungsten coil that provides uniform Joule heating to the sample area.

### Precursor Supply

The supply of growth precursors to the sample area is enabled by a gas injector mounted to the side of the microscope connected to a metal–organic chemical vapor deposition (MOCVD) system. The supply of precursors was controlled by using mass-flow controllers (MFCs).

### GaSb Nanowire Growth

In order to grow GaSb nanowires, trimethylgallium (TMGa) with a partial pressure of 1.9 × 10^–3^ Pa and trimethylantimony (TMSb) with a partial pressure of 8.6 × 10^–2^ Pa were used as precursors, with H_2_ being used as the carrier gas for both precursors. The growth was conducted at a nominal chip temperature of 420 °C. The nucleation and growth of nanowires was performed *in* situ from Au nanoparticles residing on the electron-transparent SiN_*x*_ film. More details of the growth can be found elsewhere.^7,20^

### GaSb/GaAs Heterojunction Formation

The formation of GaSb/GaAs heterojunctions was conducted using a 2-step approach. First, arsine (AsH_3_) was supplied into the growth chamber for 1 min at a partial pressure of 7.9 × 10^–2^ Pa in conjunction with TMGa and TMSb, in order to initiate the switch. After the 1 min mark, the supply of TMSb and H_2_ through the group V line of our system was ceased and AsH_3_ partial pressure was increased to 2.0 Pa. The heterojunction formation was conducted at a nominal MEMS chip temperature of 420 °C.

### Group V Line Venting

To avoid pressure build-up in the gas handling system, in addition to direct supply to the microscope, it is possible to vent precursors directly to an exhaust line leading into a scrubber.^30,32^ To minimize TMSb background in the growth chamber during GaSb/GaAs heterojunction formation, using the procedure discussed in section Kinking/Change of Growth Direction, the supply of TMSb to the microscope was stopped, and the entire group V line length was vented to the exhaust line prior to the initiation of the AsH_3_ supply. Subsequently, the valves to the exhaust line were closed.

### Residual Gas Analyzer Data

To determine the dynamic change in the vapor-phase composition during the heterostructure formation process, the partial pressures of atomic As and Sb were tracked in the residual gas analyzer.

### *In Situ* Video Processing for the Supporting Information

The enclosed *in situ* Video S1 and Video S3 were processed by binning the acquired videos of heterostructure formation 4 times (from 2048 × 2048 pixel resolution to 512 × 512 pixel resolution), whereas Video S2 was binned 2 times (from 2048 × 2048 pixel resolution to 1024 × 1024 pixel resolution). Furthermore, 20 frames were summed together to increase the signal-to-noise ratio, which also reduced temporal resolution from 20 to 1 FPS. Lastly, the *in situ* video was compressed using JPEG compression using ImageJ 2.0.0.

## Supplementary Material









## Notes

Real space TEM video of GaSb/GaAs heterojunction formation 16x (AVI)
Real space TEM and GPA video encompassing the full compositional switch of GaSb/GaAs heterojunction formation 4x (AVI)
Real space TEM and GPA video showcasing kinking due to the formation of a multilayer stack sped up 4x (AVI)
Details of the precursor and carrier gas partial pressures; nanowire morphological evolution before and after heterojunction formation; and X-ray energy dispersive spectroscopy data of nanowires (PDF)
