# Supplementary material for: In Situ Study of Axial GaSb/GaAs Nanowire Heterostructure Formation
Source: ACS Nanosci Au. 2025 Apr 15;5(3):208–16. doi: 10.1021/acsnanoscienceau.5c00015 (PMC12183588; doi:10.1021/acsnanoscienceau.5c00015)
Supplement: Supplementary file 4 [file ng5c00015_si_004.pdf]

Supporting information for: *In situ* study of axial GaSb/GaAs nanowire heterostructure formation

Mikelis Marnauza[1], Robin Sjökvist[1], Azemina Kraina[1], Daniel Jacobsson[1,2], Kimberly A. Dick[1]

*Corresponding author e-mail: kimberly.dick_thelander@chem.lu.se

[1]Centre for Analysis and Synthesis and NanoLund, Lund University, 22100 Lund, Sweden

[2]National Centre for High Resolution Electron Microscopy, Lund University, 22100 Lund, Sweden

SI-1: Precursor and carrier gas partial pressures during heterostructure formation

Table 1. Growth conditions used during the GaSb/GaAs heterostructure formation.

Notes	TMGa partial pressure, Pa	TMSb partial pressure, Pa	AsH_3 partial pressure, Pa	H_2 partial pressure, Pa
GaSb growth	3.8E-03	1.6E-01	-	5.3
Ternary growth	3.5E-03	1.5E-01	7.3E-02	5.2
GaAs growth	1.5E-03	-	2.0E+00	0.3

SI-2: Nanowire morphological evolution after heterostructure formation

Figure S1 (a) shows a high-resolution transmission electron microscope (HRTEM) image of a Au-seeded GaSb nanowire during growth. In Figure S1 (b) the same nanowire is imaged after steady-state growth of GaAs is achieved. When comparing the images it can be observed that, in addition to axial growth, the original GaSb nanowire has undergone significant overgrowth.

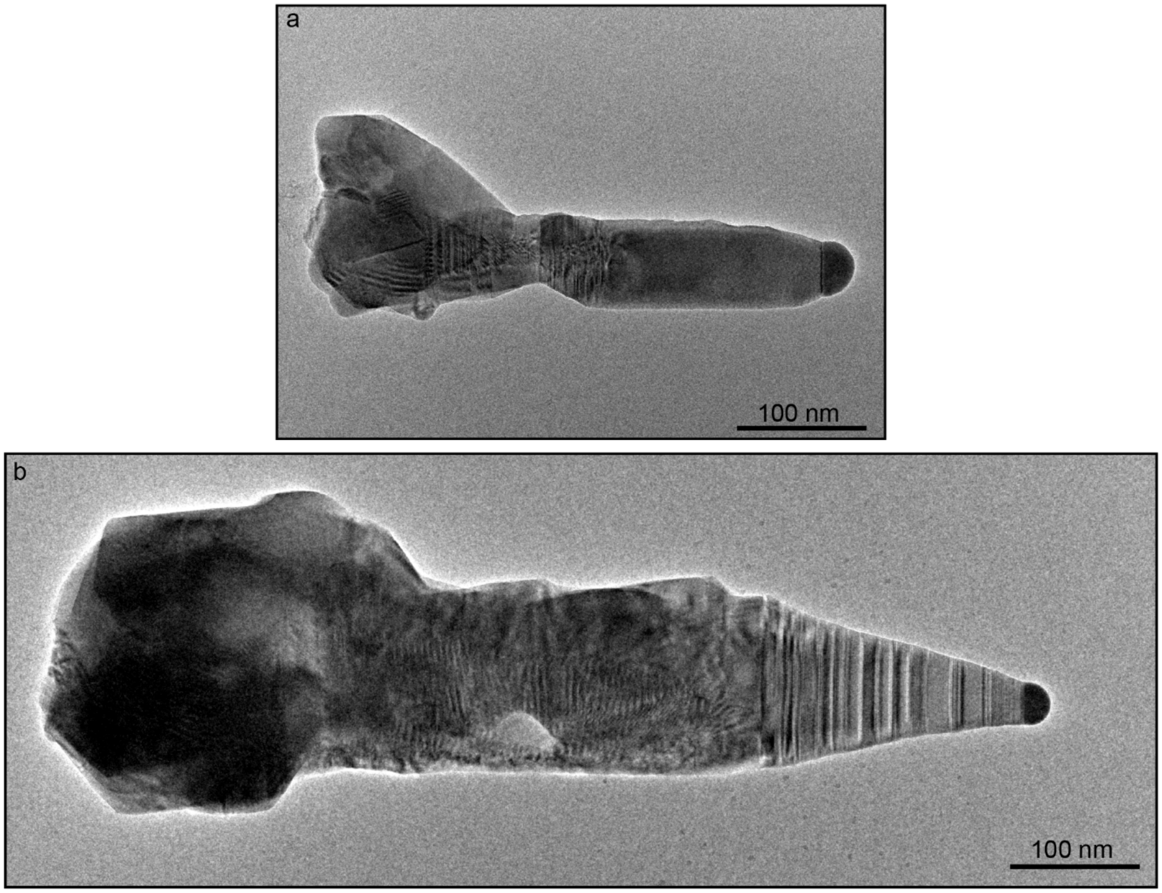

Figure S1: GaSb/GaAs nanowire heterostructure. (a) HRTEM image of a Au-seeded GaSb nanowire during growth. (b) HRTEM image of the same nanowire after steady-state of GaAs is achieved. The scale bar in both images is the same.

SI-3: X-ray energy dispersive spectroscopy

To verify that the heterostructure involves transitioning from pure GaSb to pure GaAs we carried out X-ray energy dispersive spectroscopy (XEDS) analysis. In Figure S2 (a) an HRTEM image of a GaSb nanowire is shown, where the blue circle denotes approximate size and position of the electron probe during XEDS acquisition. Similarly, in Figure S2 (b) an HRTEM image of the same nanowire after switching to GaAs is shown with the blue circle denoting approximate size and position of the electron probe during XEDS acquisition.

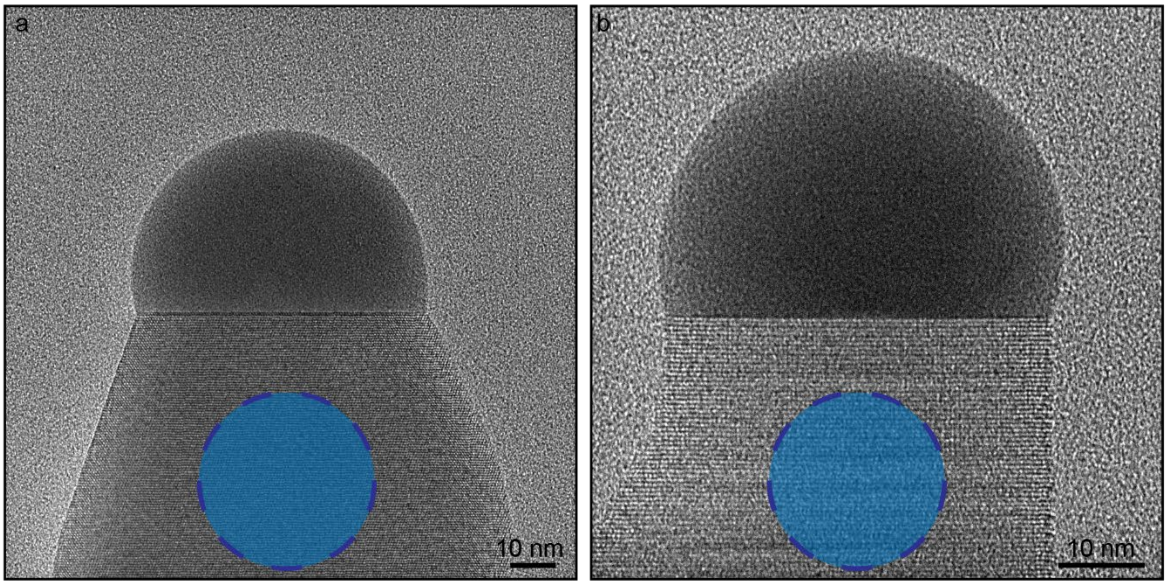

Figure S2: Example of a nanowire used to carry out compositional analysis. (a) HRTEM image of a GaSb nanowire during growth. (b) HRTEM image of the same nanowire after switching to GaAs. Blue circles in (a) and (b) panels serve to illustrate approximate position and size of the electron probe during XEDS spectra acquisition.

Quantified spectra for nanowires in Figure S2 can be viewed in Table S1. As the XEDS data was acquired during nanowire growth at 420 °C the error is expected to be ≈ 2 at.%.[1] It is evident that during GaSb nanowire growth the spectra show a departure from the expected 50:50 ratio between Ga and Sb signals, which can be attributed to the electron channelling effect, which is especially prominent when acquiring XEDS spectra in zone axis conditions.[2] Therefore, the quantified spectra show that the nanowire undergoes a full compositional change from GaSb to GaAs.

Table S1: Summary of XEDS quantification results for nanowire before (Figure S2 (a)) and after switching (Figure S2 (b)) from GaSb to GaAs.

Spectrum label	Ga at.%	As at.%	Sb at.%
Figure S2 (a) (before switching)	56.7	1.8	41.5
Figure S2 (b) (after switching)	50.1	49.3	0.6

To qualitatively examine the composition of overgrowth on the original GaSb nanowire post-growth XEDS mapping was carried out using scanning transmission electron microscopy (STEM). The high angle annular dark field (HAADF) STEM image along with XEDS maps for Ga, Sb and As are summarized in Figure S. Here it can be seen that the original GaSb nanowire is overgrown by an As-rich solid.

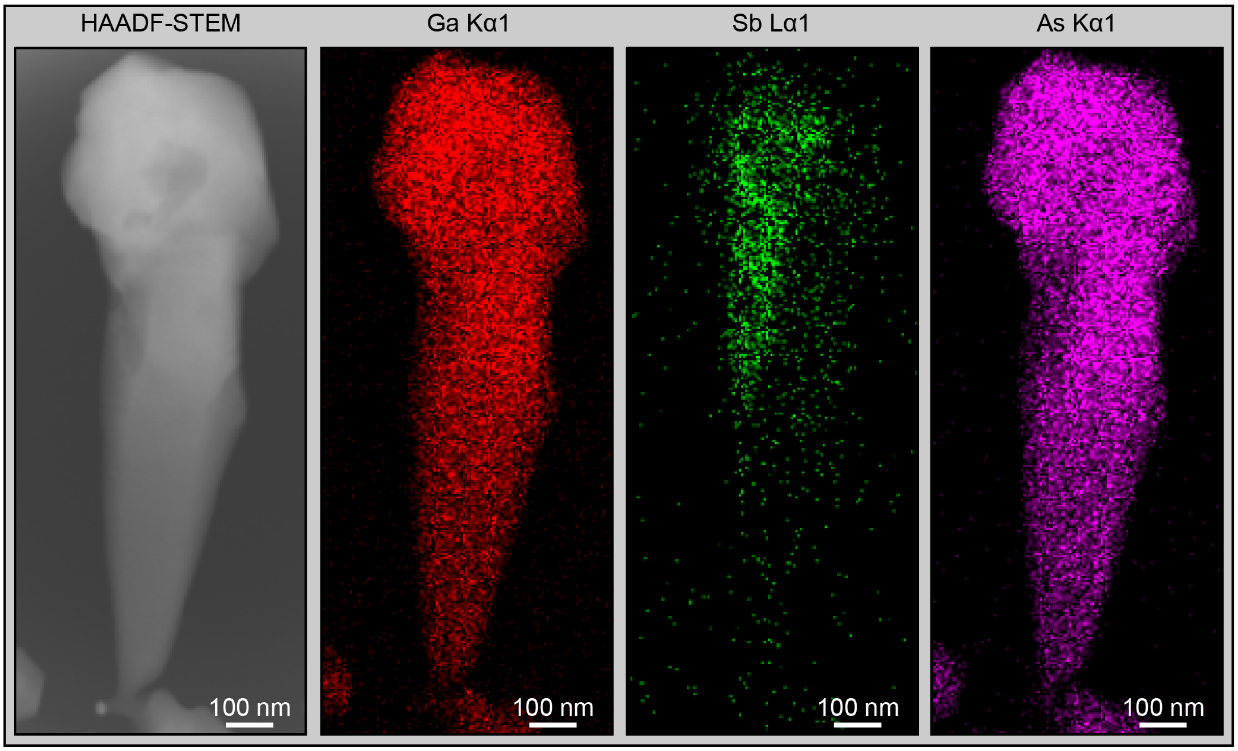

Figure S3: STEM-XEDS mapping of the GaSb/GaAs heterostructure post growth.

References

(1) Maliakkal, C. B.; Mårtensson, E. K.; Tornberg, M. U.; Jacobsson, D.; Persson, A. R.; Johansson, J.; Wallenberg, L. R.; Dick, K. A. Independent Control of Nucleation and Layer Growth in Nanowires. *ACS Nano* **2020**, *14* (4), 3719–5150. https://doi.org/10.1021/acsnano.9b09816.

(2) Ek, M.; Lehmann, S.; Wallenberg, R. Electron Channelling: Challenges and Opportunities for Compositional Analysis of Nanowires by TEM. *Nanotechnology* **2020**, *31* (36). https://doi.org/10.1088/1361-6528/ab9679.
